# Differential epitope recognition in the immunodominant staphylococcal antigen A of *Staphylococcus aureus* by mouse versus human IgG antibodies

**DOI:** 10.1038/s41598-017-08182-9

**Published:** 2017-08-15

**Authors:** Dennis G. A. M. Koedijk, Francisco Romero Pastrana, Hedzer Hoekstra, Sanne van den Berg, Jaap Willem Back, Carolien Kerstholt, Rianne C. Prins, Irma A. J. M. Bakker-Woudenberg, Jan Maarten van Dijl, Girbe Buist

**Affiliations:** 1 Department of Medical Microbiology, University of Groningen, University Medical Center Groningen, Hanzeplein 1, P.O. Box 30001, 9700 RB Groningen, The Netherlands; 2 Department of Medical Microbiology and Infectious Diseases, Erasmus University Medical Center, Rotterdam, The Netherlands; 3 Pepscan Therapeutics BV, Lelystad, The Netherlands

## Abstract

The immunodominant staphylococcal antigen A (IsaA) is a potential target for active or passive immunization against the important human pathogen *Staphylococcus aureus*. Consistent with this view, monoclonal antibodies against IsaA were previously shown to be protective against *S*. *aureus* infections in mouse models. Further, patients with the genetic blistering disease epidermolysis bullosa (EB) displayed high IsaA-specific IgG levels that could potentially be protective. Yet, mice actively immunized with IsaA were not protected against *S*. *aureus* infection. The present study was aimed at explaining these differences in IsaA-specific immune responses. By epitope mapping, we show that the protective human monoclonal antibody (humAb) 1D9 recognizes a conserved 62-residue N-terminal domain of IsaA. The same region of IsaA is recognized by IgGs in EB patient sera. Further, we show by immunofluorescence microscopy that this N-terminal IsaA domain is exposed on the *S*. *aureus* cell surface. In contrast to the humAb 1D9 and IgGs from EB patients, the non-protective IgGs from mice immunized with IsaA were shown to predominantly bind the C-terminal domain of IsaA. Altogether, these observations focus attention on the N-terminal region of IsaA as a potential target for future immunization against *S*. *aureus*.

## Introduction

*Staphylococcus aureus* can cause a wide variety of diseases and has a strong tendency of developing resistance against multiple antibiotics^1^. Methicillin-Resistant *S*. *aureus* (MRSA)- associated infections are becoming increasingly harder to treat. Therefore, a renewed focus on the development of alternative means of treatment has arisen. Whereas many infectious diseases are nowadays controlled through vaccination, *S*. *aureus* immunity has proven hard to achieve with vaccines^2–5^. As an alternative to the active immunization against *S*. *aureus* with vaccines, passive immunization with monoclonal antibodies specifically targeting *S*. *aureus* is currently explored^6–11^.

Invariantly expressed cell surface-exposed proteins are attractive potential targets for immunization, due to their high accessibility to the human immune system^12^. This focused attention on the immunodominant *S*. *aureus* antigen A (IsaA). The IsaA protein was first described in the year 2000 as an antigen recognized by IgGs from patients with sepsis caused by MRSA^13^. Subsequent, proteomic analyses of the *S*. *aureus* exoproteome revealed that the IsaA protein was invariantly produced by all investigated isolates of this pathogen^14, 15^. More specifically, IsaA is a non-covalently cell wall attached protein that is both exposed to the cell surface and secreted^13, 16, 17^. The IsaA protein has a putative soluble lytic transglycosylase domain at the C-terminus, indicating a role in peptidoglycan turnover and cell wall hydrolysis^17, 18^. Interestingly, this C-terminal active site domain is exposed to the staphylococcal cell surface while the precise localization of the N-terminal domain was so far not known^12, 18^. Further, IsaA was localized to the septal region of dividing cells, suggesting its involvement in cell growth, separation and survival^16^. In addition to its potential role in cell division, IsaA was shown to be involved in biofilm formation^17, 19, 20^.

The immunodominant nature of IsaA was confirmed by different studies showing that both IgG1 and IgG4 against this protein are present in sera from a wide range of healthy human individuals and patients^13, 21–24^. Interestingly, healthy *S*. *aureus* carriers showed significantly higher IgG levels against IsaA than non-carriers^22^. Furthermore, patients with the genetic blistering disease epidermolysis bullosa (EB), who are highly colonized with *S*. *aureus*, displayed very high IgG responses against IsaA^23^. Notably, EB patients do not frequently suffer from *S*. *aureus* bacteremia, even though their skin barrier provides only limited protection against *S*. *aureus*. Although not conclusive, these observations did suggest that the anti-IsaA antibodies and other anti-staphylococcal antibodies could perhaps be involved in the protection of EB patients against serious *S*. *aureus* infections. Furthermore, the high immunogenicity of IsaA and its ubiquitous presence in *S*. *aureus* indicated that IsaA could be a suitable target for passive immunization. This view was supported by passive immunization experiments in animal studies. Firstly, a murine monoclonal antibody (mAb) targeting IsaA was shown to be protective in mouse models of catheter-related *S*. *aureus* infection^7^, and a humanized form of this mAb mediated bacterial killing in blood samples from high risk patients^8^. In another study, we showed that a fully human monoclonal antibody (humAb) against IsaA (designated 1D9) improved the survival of mice challenged with a clinical *S*. *aureus* isolate^6^.

Intriguingly, several active immunization studies in animals also revealed high immune responses against IsaA^25–28^. Nonetheless, no protection against *S*. *aureus* infection was observed in these immunized animals. This raised the question why certain monoclonal antibodies against IsaA, such as 1D9, are at least partially protective against *S*. *aureus* infection, while high anti-IsaA IgG levels raised by animal immunization were not protective. The present study was aimed at explaining this difference by assessing the IsaA epitopes recognized by the apparently protective and non-protective antibodies. Briefly, the results show that potentially protective antibodies recognize a different epitope in IsaA than the non-protective antibodies.

## Materials and Methods

### Bioinformatics

BLAST searches were performed with default search parameters (Program BLASTP 2.3.0+, refs 29 and 30; word size 6; expect value 10; hitlist size 1000; gapcosts 11,1; matrix BLOSUM62; filter string F; genetic code 1; window size 40; threshold 21; composition-based stats 2) against the NCBI Protein Reference Sequences Database (refseq_protein; posted date: December 7, 2015 10:23 AM). Pfam sequence searches (http://pfam.xfam.org/search) were performed with default options: use E-value 1.0 cut-off^31^. Multiple sequence alignments were performed using COBALT^32^ with default parameters (alignment parameters: gap penalties -11, -1; end-gap penalties -5, -1; CDD Parameters: Use RPS BLAST on; Blast E-value 0.003; find conserved columns and recompute on; query clustering parameters: use query clusters on; word size 4; max cluster distance 0.8; alphabet regular). Visualizations of HMM logos (representing both sequence alignments and profile hidden Markov models) were obtained using Skylign (http://skylign.org)^33^ with the following parameters: alphabet: AA; letter height: information content above background; alignment processing: HMM - remove mostly-empty columns. Predicted protein masses (kDa) and isoelectric points (pI) were obtained using the Expasy compute pI/Mw tool (web.expasy.org/compute_pi/). Data from Western blots were quantified by ImageJ software (available via http://rsbweb.nih.gov/ij/).

### Bacterial strains and growth conditions

Bacterial strains and plasmids that were used in this study are listed in Table 1. Staphylococcal strains were grown at 37 °C, 250 rpm (0.87 × *g*) in Tryptone Soy Broth (TSB; Oxoid, Hampshire, UK). *L*. *lactis* strains were grown at 30 °C in M17 broth (Oxoid Limited) or on M17 plates with 1.5% agar and 0.5% glucose (w/v), supplemented with chloramphenicol (5 µg/ml) for plasmid selection. For *in vivo* studies the *S*. *aureus* clinical isolate P^15^ was grown in Brain Heart Infusion broth (BHI; Becton Dickinson, Breda, The Netherlands).

**Table 1:** Bacterial strains and plasmids used in this study.

Strain or plasmid	Relevant phenotype(s) or genotype(s)	Source or reference
**Strains**		
*L*. *lactis* PA1001	MG1363 *pepN*::*nisRK* allows nisin-inducible expression, Δ*acmA* Δ*htrA*	46
*S*. *aureus* NCTC8325-4	NCTC8325 cured of ϕ11, ϕ12, and ϕ13	47
*S*. *aureus* isolate P	Community-acquired MSSA strain from blood of a septic patient	15
*S*. *aureus* MA12 Δ*isaA*	*S*. *aureus* MA12 *isaA* mutant	7
*E*. *coli* BL21DE3	Allows IPTG-inducible expression of P_T7_	Novagen
*S*. *aureus* Newman Δ*spa*Δ*sbi*	*spa sbi* mutant	36
*S*. *aureus* isolates D, E, F, G, H	Community- and hospital acquired clinical isolates collected in the University Medical Center Groningen over a 4.5-year period	15
**Plasmids**		
pET24d::*isaA*::*his*_6_	*Kan* ^*R*^, pET24d containing *isaA* with C-terminal *his*_6_	34
pNG4110	Cm^R^, pNG400, containing P_nisA_, SS_usp45_, *his*_6_, *Bam*HI/*Eco*RI-*Xba*I/*Not*I cloning sites	48
pNG4210	pNG400 containing *Bam*HI/*Eco*RI-*Xba*I/*Not*I cloning sites, *his*_6_	48
pNG4110::*isaA*	pNG4110 encoding residues 30–233^a^ from *S*. *aureus* NCTC8325 IsaA	This study
pNG4110::*isaA*-N	pNG4110 encoding residues 30–145 from IsaA	This study
pNG4110::*isaA*-N1	pNG4110 encoding residues 30–91 from IsaA	This study
pNG4110::*isaA*-N2	pNG4110 encoding residues 92–145 from IsaA	This study
pNG4110::*isaA*-C	pNG4110 encoding residues 146–233 from IsaA	This study
pNG4110::*isaA*-C1	pNG4110 encoding residues 146–189 from IsaA	This study
pNG4110::*isaA*-C2	pNG4110 encoding residues 190–233 from IsaA	This study
pNG4210::*isaA*	pNG4210 encoding residues 30–233 from IsaA	This study
pNG4210::*isaA*-N	pNG4210 encoding residues 30–145 from IsaA	This study
pNG4210::*isaA*-N1	pNG4210 encoding residues 30–91 from IsaA	This study
pNG4210::*isaA*-N2	pNG4210 encoding residues 92–145 from IsaA	This study
pNG4210::*isaA*-C	pNG4210 encoding residues 146–233 from IsaA	This study
pNG4210::*isaA*-C1	pNG4210 encoding residues 146–189 from IsaA	This study
pNG4210::*isaA*-C2	pNG4210 encoding residues 190–233 from IsaA	This study

*Kan* ^*R*^, *kanamycin resistance gene; Cm* ^*R*^, *chloramphenicol resistance gene; P*_*T7*_, *IPTG inducible T7-promoter; P*_*nisA*_, *nisin inducible promoter; his*_6_, *6 histidine-tag; SS*_*usp45*_, *signal sequence of usp45*; ^a^position of amino acid residues (aa) in IsaA sequence of *S. aureus* NCTC8325 (YP_501340).

### Construction of expression plasmids for the production of IsaA and its derivatives

PCR primers for the construction of IsaA protein-expressing plasmids are shown in Supplementary Table 1. DNA was isolated with the Genelute bacterial genomic DNA kit (Sigma-Aldrich, Zwijndrecht, The Netherlands). PCR was performed with the Phusion Hot Start II polymerase (Thermo Fisher Scientific, Wilmington, Delaware USA) using genomic DNA of *S*. *aureus* NCTC8325 as a template as described before^34^. The PCR fragments purified using the High Pure PCR purification kit (Analytic Jena, Jena, Germany) were cleft with *Bam*HI and *Not*I restriction enzymes (New England Biolabs, Ipswich, USA) and ligated to *Bam*HI/*Not*I-linearized vector DNA using T4 DNA Ligase (New England Biolabs). Of note, PCR products obtained with primer combinations including a reverse primer with a stop codon (F1/R2) were inserted into plasmids pNG4110, and PCR products obtained with reverse primers lacking a stop codon (F1/R1) were inserted into plasmid pNG4210. The resulting plasmids were transferred to electrocompetent *L*. *lactis* PA1001 as described before^35^. All plasmids were verified by sequencing (Eurofins MWG Operon, Ebersberg, Germany).

### HumAb production and labeling

The humAb 1D9 directed against *S*. *aureus* IsaA was produced as described before^6^. Briefly, Expi293 cells were transiently transfected with plasmids encoding the H and K fragments of 1D9 (Expi293 Expression System, Life Technologies). Secreted 1D9 antibodies were isolated from the cell culture medium by Protein A column purification (HiTrap Protein A HP, GE Lifesciences) followed by desalting (HiTrap Desalting, GE Lifesciences) according to the manufacturer’s protocol. 1D9 F(ab’)_2_ nanobodies were derived from the complete 1D9 humAb using the FragIT kit (Genovis, Sweden) following the manufacturer’s protocol. The complete 1D9 antibody or the respective 1D9 F(ab’)_2_ nanobodies were labeled with IRDye 800CW (LI-COR Biosciences, Bad Homburg, Germany) by incubation with 20 µg of this dye per mg of protein in PBS (pH 8.5, 2 h, room temperature). The labeled antibodies were isolated and desalted as described above, and they were stored in the dark at 4 °C.

### Protein expression, purification and detection of IsaA and its subdomains

The production and isolation of IsaA-His_6_ from *E*. *coli* BL21DE3 (pET24d::*isaA*::*his*_6_) was performed as described previously^6^. Expression of IsaA and its derivatives in *L*. *lactis* PA1001 was induced with nisin (3 ng/ml, Sigma-Aldrich, St. Luis, MO) at an Optical Density at 600 nm (OD_600_) of ~0.5. After continued overnight incubation at 30 °C, cells were separated from the growth medium by centrifugation. Protein samples from both fractions were prepared as described before^34^. All proteins obtained were analyzed by Lithium Dodecyl Sulphate (LDS)-PAGE (NuPAGE gels, Life Technologies), and visualized either by protein staining (Simply Blue^TM^ Safe Staining, Life Technologies) or by Western blotting on nitrocellulose membranes (Protan nitrocellulose transfer paper, Whatman, Germany). Immunodetection of particular proteins was achieved with the following antibodies or sera: mouse anti-His tag (Life Technologies), rabbit anti-IsaA (kindly provided by N. Sakata)^16^, sera from mice immunized with IsaA, rabbit anti-SceD (kindly provided by S. Foster)^17^ or the IRDye 800 CW-labeled 1D9 anti-IsaA humAb^6^. The binding of unlabeled antibodies was visualized with fluorescently labeled goat anti-human, goat anti-mouse or donkey anti-rabbit IRDye 800 CW-labeled secondary antibodies (LI-COR Biosciences, Lincoln, NE. USA), using the Odyssey Infrared Imaging System (LI-COR Biosciences).

### Cell wall extraction and binding experiments

Crude cell wall extracts were obtained from overnight cultures of *S*. *aureus* MA12 Δ*isa*A or *S*. *aureus* Newman Δ*spa*Δ*sbi*. To this end, cell pellets were resuspended in demineralized water with 0.1 µm glass beads (Biospec Products, Bartlesville, USA) and disrupted in a Precellys 24 homogenizer (Bertin Technologies, France), followed by centrifugation at 4000 rpm (1520 × *g*) for 6 min. Recovered supernatants were centrifuged for 15 min at 14000 rpm (18620 x *g*) and pellets containing crude cell wall extracts were resuspended in demineralized water and stored at 4 °C. Non-covalently bound cell wall proteins were extracted from *S*. *aureus* Newman Δ*spa*Δ*sbi* cells by a 10 min incubation with 1 M potassium thiocyanate (KSCN) as described previously^36^.

Growth medium fractions of overnight nisin-induced cultures expressing IsaA protein fragments were precipitated with 10% TCA. The resulting protein pellets were resuspended in 4-fold diluted phosphate buffered saline with 0.1% Tween 20 (PBS-T), and proteins were analyzed by LDS-PAGE. For rebinding experiments, crude cell wall extracts were incubated with IsaA protein fragments (obtained as described above) at room temperature for 10 min and, thereafter, the binding of the IsaA fragments was assessed by LDS-PAGE and Western blotting.

### Near Infrared Microscopy

Aliquots of 1 ml of bacterial cultures (OD_600_ of 1) in TSB were collected, washed twice with PBS, and resuspended in 1 ml of PBS. Next, ~10^10^ cells thus obtained were incubated with 0.65 mg/ml IRDye 800CW-labeled 1D9 or 1D9 F(ab’)_2_ for 1 h at room temperature. After washing 3 times with PBS, cells were spotted on poly-L-lysine-coated glass slides (Sigma Aldrich) and inspected using a Leica DM5500B epifluorescence microscope equipped with an 800 nm filter block. Images were captured with a Leica DFC365FX camera using a 63× objective (Leica Microsystems BV, The Netherlands).

### Epitope mapping with peptide arrays

To determine which epitopes of *S*. *aureus* IsaA were recognized by the investigated anti-IsaA IgGs, libraries of linear 15-mer IsaA-specific peptides were synthesized with an overlap on a solid support (Pepscan), as previously described^37, 38^. Next, the peptide libraries were probed with heat-inactivated mouse sera, humAb 1D9, or rabbit anti-IsaA in a dilution of 1:1000. Bound IsaA-specific antibodies were detected with a secondary HRP-conjugated antibody (Southern Biotech, Birmingham, USA) and 2,2′-azino-bis(3-ethylbenzothiazoline-6-sulphonic acid) (Sigma-Aldrich). A charge-coupled device camera was used to record the absorbance at 405 nm.

### Immunization of mice with IsaA

Specified pathogen-free female BALB/cBYJ mice were obtained from Charles River (Saint-Germain-sur-l’Arbresle, France). Mice were treated and selected as described previously^27^. Purified IsaA-His_6_ was emulsified 1:1 with TiterMax Gold adjuvant (Sigma-Aldrich). Mice were immunized subcutaneously in the flank with 100 μL formulated vaccine on days -28, -21, and -14 (25 μg of antigen). Control mice received 100 μL PBS emulsified with adjuvant. At day -1, blood was withdrawn from the tail artery. Sera were examined by ELISA using ELISA plates (Greiner Bio-One B.V, Alphen aan den Rijn, the Netherlands), coating, blocking, hybridization and detection procedures as described previously^27^. Immunized mice (n = 6 immunized mice, n = 11 placebo-immunized mice) were challenged on day 0 by intravenous inoculation of 100 μL of *S*. *aureus* isolate P (3 × 10^5^ CFU) as described previously^27^. Discomfort and animal survival rate over 14 days after infection were monitored. For discomfort score, clinical signs of illness in each mouse were evaluated at least twice daily as described before^27^.

### Ethics statements

Blood donations from EB patients were collected under the approval of the medical ethics committee of the University Medical Center Groningen (approval no. NL27471,042,09) upon written informed patient consent, and with adherence to the Helsinki Guidelines^23^. The required written informed consent was obtained from all EB patients and healthy volunteers included in the present studies. The Institutional Animal Care and Use Committee of the Erasmus University Medical Centre Rotterdam approved the present protocols for animal experiments (permit number: EMC2694). All animal experiments were performed in accordance with the rules laid down in the Dutch Animal Experimentation Act and the Directive 2010/63/EU on the protection of animals used for scientific purposes.

## Results

### IsaA contains 2 conserved domains

**Figure 1:**
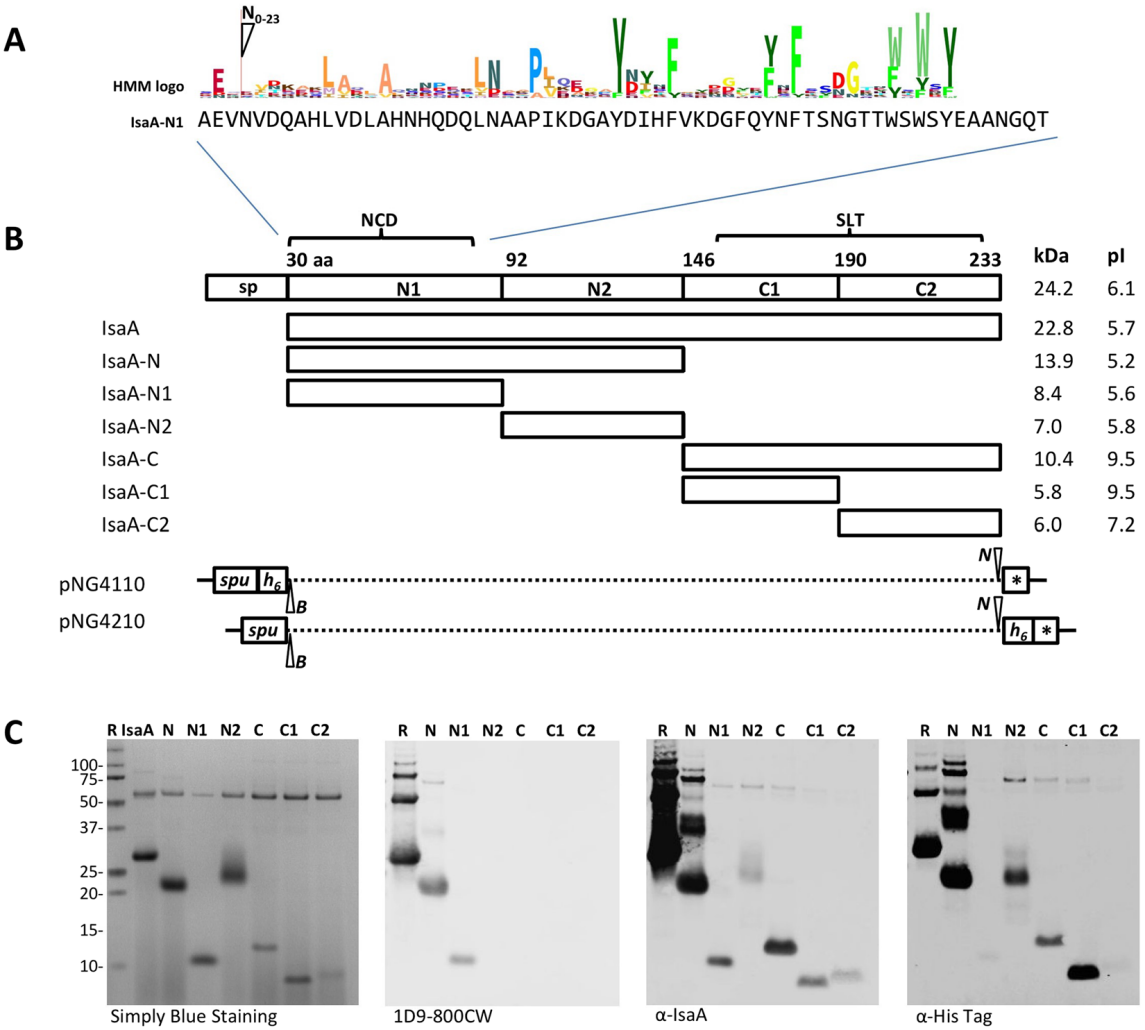
Schematic representation of IsaA subdomains, their separate expression and immunodetection. (**A**) The N-terminal conserved domain (NCD) of IsaA (residues 30–84) as identified by BLASTP searches with the N-terminal region of IsaA (residues 30–145; see also Supplementary Fig. 1). (**B**) The mature IsaA, its N-terminal (IsaA-N, IsaA-N1, IsaA-N2) or C-terminal (IsaA-C, IsaA-C1, IsaA-C2) fragments were expressed in *L*. *lactis* PA1001 and secreted as N-terminal or C-terminal His-tag fusions using the lactococcal signal peptide of Usp45 (*spu*). The C-terminal part of IsaA contains a soluble lytic transglycosylase (SLT) domain (pfam01464). In the schematic representations of the expression vectors pNG4110 and pNG4210, used to produce IsaA or its subdomains, the positions of the cleavage sites for the restriction enzymes *Bam*HI (*B*) and *Not*I (*N*), and the N-terminal or C-terminal His-tags (*h*_6_) are indicated. Molecular weights (kDa) of IsaA and its subdomains and their isoelectric points (pI) are indicated. sp, IsaA signal peptide; *Stop codon provided within the expression vectors pNG4110 and pNG4210. (**C**) LDS-PAGE analysis of growth medium fractions of nisin-induced *L*. *lactis* cultures producing the C-terminally His-tagged IsaA protein or IsaA fragments. The His-tagged proteins are visualized either by Simply Blue gel-staining, or by Western blotting with the humAb 1D9–800CW, the polyclonal α-IsaA rabbit antibodies, or α-His-tag antibodies. Molecular weights (kDa) of the standard proteins are indicated on the left.

As a first approach towards the identification of antigenic epitopes in IsaA, we performed a bioinformatics analysis of the domain structure of this protein. Inspection of the amino acid sequence of IsaA using Pfam only showed the presence of the known soluble lytic transglycosylase domain (SLT, pfam01464) in the C-terminus (Fig. 1B). Subsequent BLASTP searches against the NCBI Protein Reference Sequences Database using the N-terminus of IsaA (residues 30–145) revealed another conserved domain ranging from residues 30 to 84. A following Position-Specific iterated BLAST search with this region of IsaA showed that this domain is exclusively present in the N-termini of 391 proteins, primarily the IsaA and SceD proteins from *S*. *aureus* and other staphylococcal species (Fig. 1A and Supplemental Fig. 1). In what follows, we refer to this N-terminal Conserved Domain as the NCD domain of IsaA.

### IsaA is non-covalently bound to the staphylococcal cell wall

**Figure 2:**
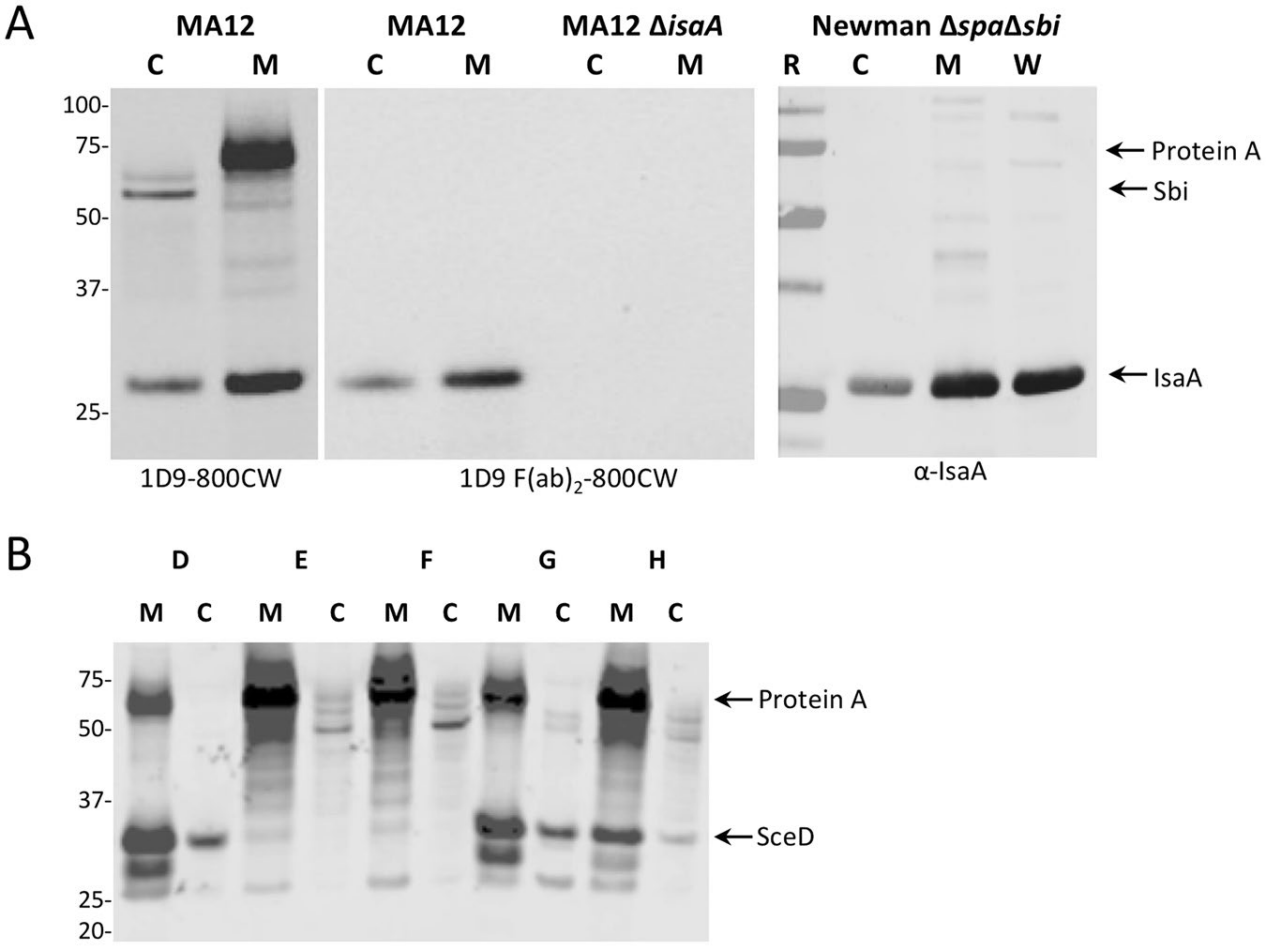
Cell-associated and secreted forms of IsaA and SceD. (**A**) *S*. *aureus* MA12, *S*. *aureus* MA12 Δ*isaA* and *S*. *aureus* Newman Δ*spa*Δ*sbi* were grown overnight. Subsequently, cells (C) were separated from the growth medium (M) by centrifugation and proteins in the respective fractions were separated by LDS-PAGE. In addition, non-covalently cell wall-associated proteins (W) were extracted from *S*. *aureus* Newman Δ*spa*Δ*sbi* and separated by LDS-PAGE. The presence of IsaA was subsequently visualized by Western blotting using humAb 1D9-800CW, hum Ab 1D9 F(ab)_2_-800CW or the polyclonal rabbit antibody α-IsaA. Molecular weights (kDa) of marker proteins are shown on the left and the positions of Protein A, Sbi and IsaA are indicated on the right. (**B**) Cellular (**C**) and secreted proteins in the growth medium (M) of the clinical *S*. *aureus* isolates (**D**,**E**,**F**,**G** and **H**) were separated by LDS-PAGE and detected by Western blotting using an α-SceD polyclonal antibody. Molecular weights (kDa) of marker proteins are shown on the left and the positions of protein A and SceD are indicated on the right.

**Figure 3:**
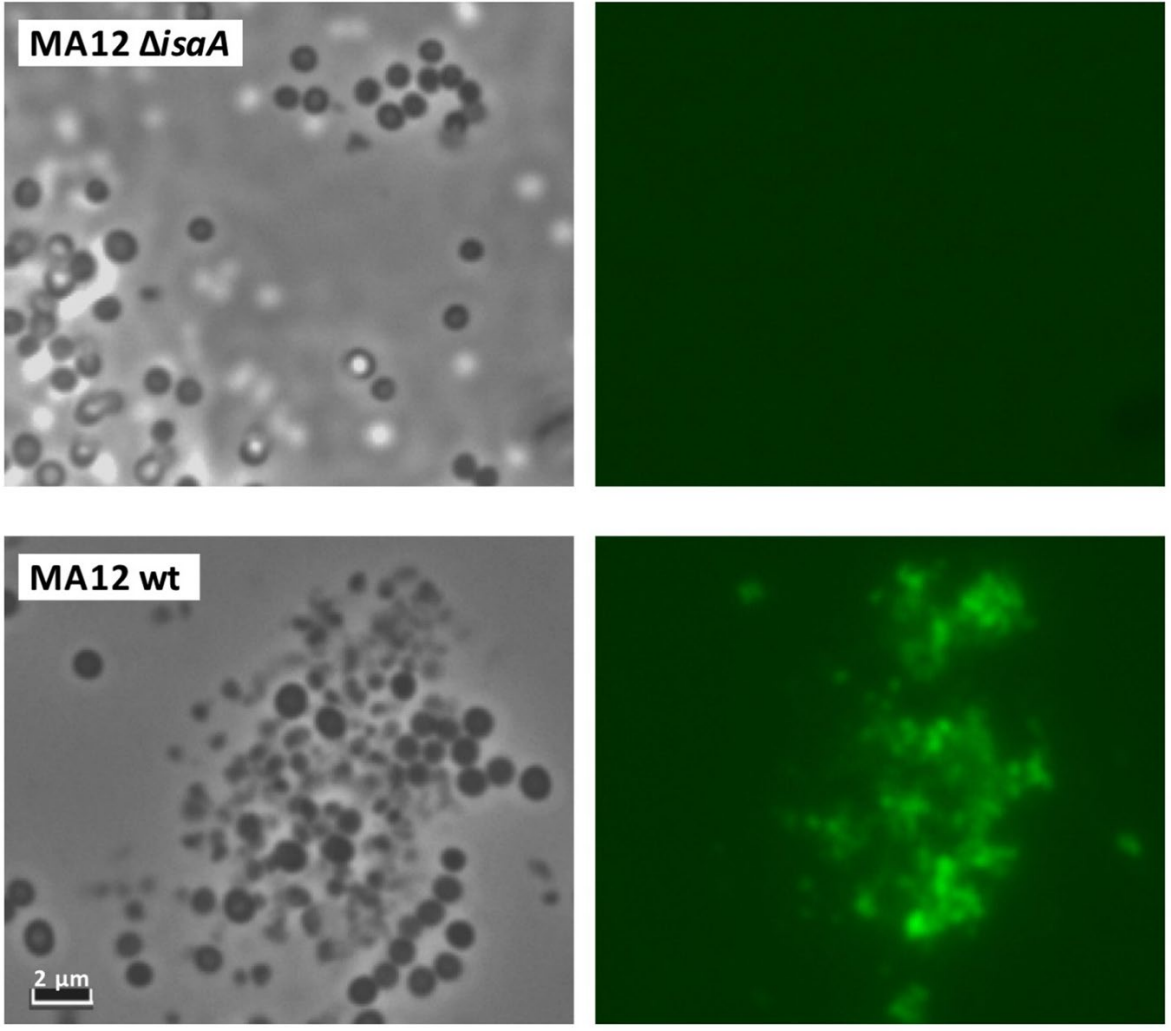
Exposure of IsaA on the *S*. *aureus* cell surface. Phase contrast (left panels) and near-infrared fluorescence (right panels) microscopic images of overnight cultured *S*. *aureus* cells treated with 1D9 F(ab)_2_-800 CW. Top panels, *S*. *aureus* MA12 Δ*isaA* cells; bottom panels wild-type (wt) *S*. *aureus* MA12 cells.

To investigate the subcellular localization of IsaA, several experiments were performed. Fractionation and subsequent Western blotting analyses of *S*. *aureus* MA12, MA12Δ*isaA* and Newman Δ*spa*Δ*sbi* showed that IsaA is both retained in the cell fraction and released into the culture medium (Fig. 2A). Furthermore, extraction of non-covalently cell wall-bound proteins from *S*. *aureus* Newman Δ*spa*Δ*sbi* cells with KSCN resulted in the release of IsaA from the cells (Fig. 2A, lane labelled W). As KSCN is a chaotrope, the release of IsaA from the cells implies that this protein is associated with the cell wall through non-covalent cell wall interactions based on hydrogen bonds, van der Waals forces, or hydrophobic interactions (Fig. 2A). In contrast to IsaA, the IsaA paralogue SceD is mostly secreted into the growth medium by clinical *S*. *aureus* isolates that produce this protein (Fig. 2B). Of note, the full size 1D9–800CW antibody also binds to the IgG binding proteins Spa (protein A) and Sbi whereas, as expected, 1D9 F(ab’)_2_-800CW nanobodies do not bind to these proteins since they lack the Fc moiety needed for Spa- and Sbi-binding (Fig. 2A). Consistent with the Western blotting data, fluorescence microscopy showed that 1D9 F(ab)_2_-800CW binds to whole cells of the wild-type *S*. *aureus* MA12 strain, but not to cells of *S*. *aureus* MA12 Δ*isaA* (Fig. 3).

**Figure 4:**
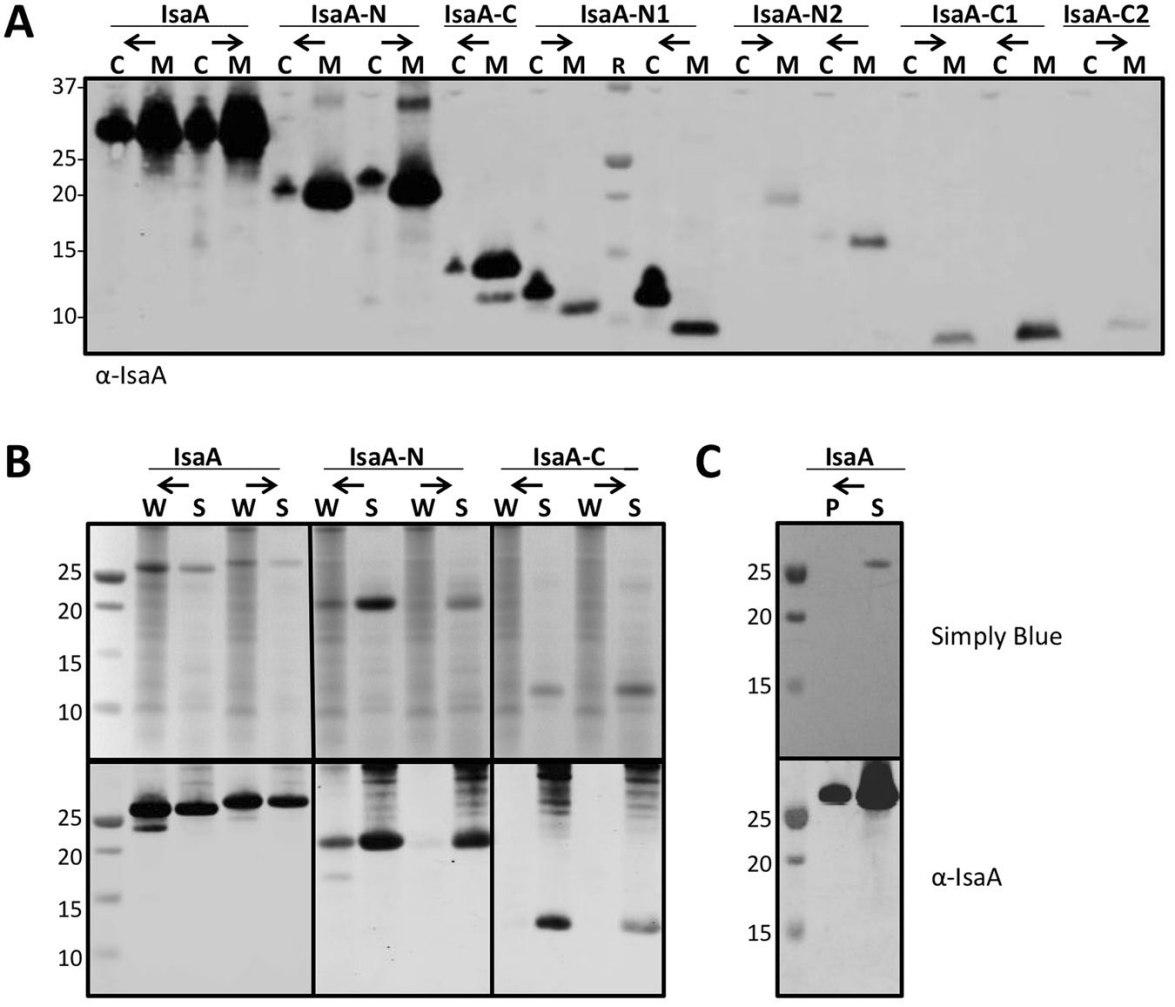
Cell wall association of IsaA-derived protein fragments. (**A**) IsaA, its N-terminal (IsaA-N, IsaA-N1, IsaA-N2) and C-terminal (IsaA-C, IsaA-C1, IsaA-C2) fragments were expressed in *L*. *lactis* PA1001 with the Usp45 signal peptide for export from the cytoplasm and an N-terminal (←) or C-terminal (→) His-tag (see Fig. 1B). Next, cells (C) were separated from the growth medium (M) by centrifugation and the presence of full-size IsaA or its fragments in the respective fractions was analyzed by Western blotting using α-IsaA polyclonal rabbit antibodies. (**B**) The rebinding potential of secreted IsaA or IsaA-derived fragments to the *S*. *aureus* cell wall was assessed using full-size IsaA or IsaA-derived fragments (as described in **A**) and crude cell wall extracts from *S*. *aureus* MA12 Δ*isa*A as described in the Materials and Methods section. Upon incubation for 10 min, the cell walls were pelleted by centrifugation and proteins in the pelleted cell wall (W) and supernatant (S) fractions were analyzed by LDS-PAGE and Simply blue staining (upper panels) or Western blotting using α-IsaA polyclonal rabbit antibodies (lower panels). (**C**) As a control, IsaA was incubated without the crude cell wall extract from *S*. *aureus* MA12 Δ*isaA* and upon incubation centrifuged. Pellet and supernatant fractions were processed as described in (**B**). The molecular weights (kDa) of marker proteins are indicated on the left.

To further investigate the potential of IsaA for cell wall binding, the mature protein and its N- and C-terminal fragments were expressed in *L*. *lactis* using vectors pNG4110 and pNG4210 as schematically represented in Fig. 1B. Specifically, this resulted in the nisin-inducible production of: the N-terminal conserved domain (N; residues 30–145), the N-terminal sub-domains N1 (residues 30–91) and N2 (residues 92–145), the C-terminal domain with the SLT active site domain, (C; residues 146–233), and the C-terminal fragments C1 (residues 146–189) and C2 (residues 190–233). These fragments were expressed either with a C-terminal His-tag or with an N-terminal His-tag. Further, all His-tagged fragments were attached to an N-terminal signal peptide, allowing their facile purification from the growth medium of *L*. *lactis* (Fig. 1C, simply blue staining panel and Western blot panel with α-His-tag antibodies). Of note, for unknown reasons the N and N2 fragments of IsaA had a lower mobility on the denaturing LDS-PAGE gels than expected based on their molecular weight. Analysis of the whole-cell and growth medium fractions of the *L*. *lactis* cultures expressing the different fragments or the full-size IsaA protein showed that mature IsaA and the N, N1 and C fragments can be detected both in the cells and the growth medium (Fig. 4A). The localization of these fragments was not affected by the presence of the His-tag at the N- or C-termini. In case of the N1 fragments most of the protein was present in the cells, suggesting that this IsaA moiety is of particular relevance for cell association. Conversely, relatively low amounts of the C fragment were localized to the cell fraction. Importantly, the N2, C1 and C2 fragments were exclusively detected in the growth medium fraction, suggesting that they have no important role in cell association (Fig. 4A).

As the above cell association analysis was performed with the heterologous organism *L*. *lactis*, a cell wall rebinding analysis was performed using crude cell wall preparations of *S*. *aureus* MA12 Δ*isaA* to verify the cell wall binding capabilities of IsaA. This approach is based on the notion that non-covalently cell wall-bound proteins that have been released into the growth medium can in principle rebind to the *S*. *aureus* cell wall. In these experiments, we focused on *S*. *aureus* cell wall rebinding of the full-size IsaA, or the N or C fragments of IsaA, as purified from the growth medium of *L*. *lactis*. Upon incubation with the cell wall preparations, the cell walls were pelleted and the presence of IsaA (fragments) in the pellet and supernatant fractions was assessed by Western blotting. As shown in Fig. 4B, the full-size IsaA and the N fragment were capable of rebinding to the *S*. *aureus* cell wall, albeit that rebinding of the N fragment was less effective depending on the position of the His-tag. In contrast, the C fragment of IsaA did not rebind to the *S*. *aureus* cell wall. For control, the assay was performed also in the absence of cell wall extract. As presented in Fig. 4C, Simply Blue gel staining showed that hardly any IsaA was pelleted in the absence of cell wall extract, although a relatively small amount of pelleted IsaA was detectable by Western blotting. The latter probably represents a small amount of aggregated IsaA or IsaA bound to the tube. Altogether, the present observations show that IsaA has a modular structure composed of a conserved N-terminal domain and an active site domain in the C-terminus. Both domains are involved in effective binding of full-size IsaA to the *S*. *aureus* cell wall, albeit that the N-terminal domain can also bind to the cell wall in absence of the C-terminal domain.

### HumAb 1D9 binds to the NCD domain of IsaA

**Figure 5:**
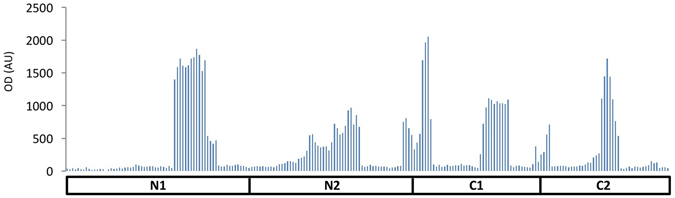
Antibody reactivity against IsaA epitopes measured by pepscan-ELISA. Bar chart with relative fluorescence signals as detected upon binding of α-IsaA polyclonal rabbit antibodies to 15-mer peptides with 14 residue overlap. The peptides are ordered on the x-axis according to the position of their N-terminal residue in the overall IsaA amino acid sequence. The relative positions of the IsaA fragments N1, N2, C1 and C2 are indicated. The y-axis shows the OD (in AU).

The potentially different functions of the N and C domains of IsaA in cell wall binding and transglycosylase activity raised the question, which epitope(s) in IsaA need(s) to be recognized for a protective antibody response? As a first approach to answer this question, we assessed the binding site of our humAb 1D9 in IsaA using the different fragments of this protein expressed in *L*. *lactis*. As demonstrated by Western blotting, humAb 1D9 labeled with IRDye800CW binds effectively to the IsaA fragments N and N1, but not to the fragments N2, C, C1 and C2 (Fig. 1C). This shows that the epitope recognized by 1D9 resides within the first 62 residues of the mature IsaA protein. By contrast, all tested fragments of IsaA were bound by the polyclonal antibodies (α-IsaA) raised in a rabbit^16^ (Fig. 1C). To further narrow down the epitope recognized by 1D9, we applied the PepScan technology^37, 38^, where antibody binding to an array with linear 15-mer peptides representing the entire IsaA protein was tested. Unexpectedly, 1D9 did not bind to this array (not shown), while the polyclonal rabbit α-IsaA antibodies did bind to the array (Fig. 5). In fact, the profile of polyclonal α-IsaA binding to the peptide array mirrored the results obtained by Western blotting, where the N, N1, N2, C, C1 and C2 fragments of IsaA were all shown to bind the polyclonal antibodies (Fig. 1C). Taken together, these findings show that humAb 1D9 binds to an epitope within the conserved NCD domain of IsaA, and that this epitope is not represented in the array of linear 15-mer peptides. The latter finding suggests that 1D9 binding to IsaA relates to a feature with a particular secondary or tertiary structure.

### Distinction of IsaA epitopes recognized by human sera and sera from IsaA-immunized mice

**Figure 6:**
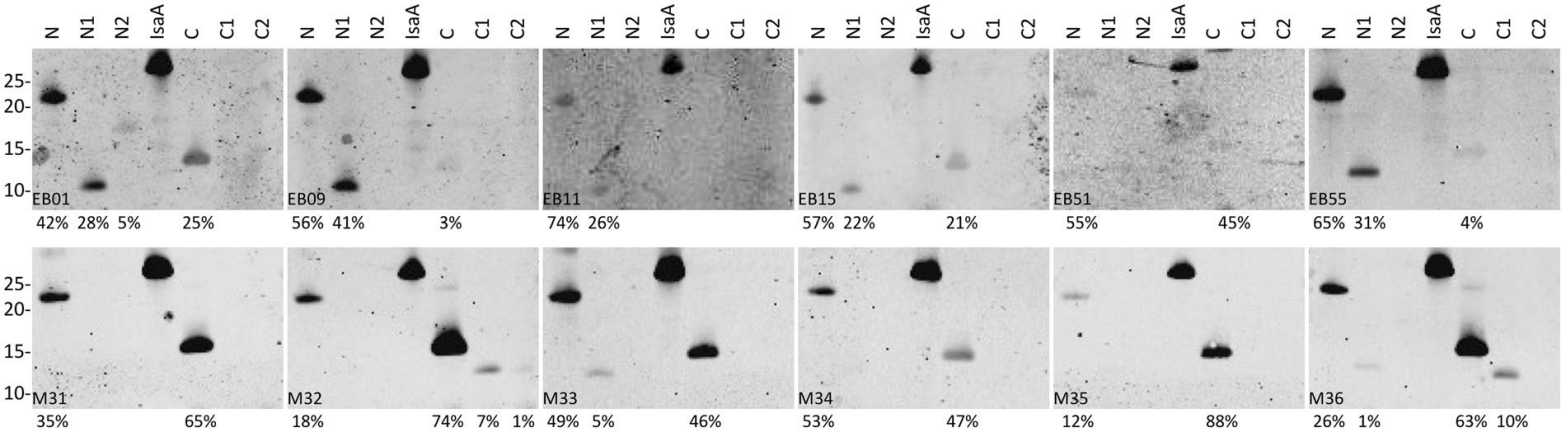
Differential binding of human and murine IgGs to IsaA-derived fragments. Proteins in the growth medium fractions of *L*. *lactis* PA1001 producing the full-size IsaA or N-terminal (N, N1, N2) or C-terminal (C, C1, C2) fragments of IsaA were separated by LDS-PAGE. Next, a Western blotting analysis was performed using sera from EB patients (EB01, EB09, EB11, EB15, EB51 and EB55) or sera from the mice immunized with IsaA (M31, M32, M33, M34, M35 and M36). The molecular weights (kDa) of marker proteins are indicated on the left. The intensities of detected bands were assessed with ImageJ, and the relative intensity for each band is indicated below each blot as the percentage of the total intensity determined for full-size IsaA *plus* the N-terminal (N, N1, N2) and C-terminal (C, C1, C2) fragments.

Our previous mouse immunization study with an octavalent anti-*S*. *aureus* vaccine including IsaA showed that antibodies against IsaA were not protective^27^. This previous conclusion was verified by immunizing mice with IsaA only, which showed that high IsaA-specific IgG titers did not protect mice against a challenge with the methicillin-sensitive *S*. *aureus* (MSSA) isolate P (Supplementary Figs 2 and 3). To determine whether the IsaA epitopes recognized by potentially protective IsaA-specific IgGs from six EB patients, and six non-protective IsaA-specific IgGs from immunized mice are similar or different, we employed a Western blotting approach. The results in Fig. 6 show that all tested sera of mice and men contained IgGs recognizing the full-size IsaA and the IsaA fragment N. All sera from mice contained IgGs recognizing fragment C, whereas this was significantly less common for the sera from EB patients. None of the tested sera contained IgGs recognizing fragment C2, and only two of the tested mouse sera contained IgGs recognizing fragment C1. Intriguingly, a distinguishing feature of the EB patient sera was that, with exception of the EB patient serum 51, all contained significant levels of IgGs against the N1 fragment (between ~22 to 41% of the total signal). Overall, this suggests that the IsaA-specific IgGs of EB patients preferentially recognize the N1 fragment of IsaA, similar to the protective humAb 1D9, whereas the non-protective IsaA-specific IgGs of immunized mice preferentially recognize the C fragment of IsaA (Fig. 6).

## Discussion

The aim of this study was to localize immunogenic epitopes in the IsaA protein of *S*. *aureus* and to correlate the recognition of these epitopes by particular IgGs to protection against *S*. *aureus* infection. The results show that IsaA-specific human IgGs predominantly target a conserved N-terminal domain in IsaA, while IsaA-specific murine IgGs predominantly target the conserved C-terminal domain of IsaA.

Previous studies have shown that the C-terminal part of IsaA contains a soluble lytic transglycosylase (SLT) domain (Pfam 06737) that is conserved also in the SceD protein of *S*. *aureus*. This SLT domain is able to cleave peptidoglycan, and impacts on the clumping and separation of *S*. *aureus* cells^17^. In contrast, a possible function of the N-terminal region of IsaA was so far not known. Of note, our present data show that this N-terminal region contains a domain (NCD) that is well conserved in the IsaA and SceD proteins of a wide range of *Staphylococcus* species. Using proteomics approaches, it was previously shown that IsaA and SceD both are non-covalently cell wall-bound proteins^18, 39–41^. As it is known that most peptidoglycan hydrolases are composed of a catalytic site domain and a cell wall binding domain^42^, we hypothesized that the NCD domain could contribute to cell wall binding. Indeed, separate expression of the different regions of IsaA showed that the N-region, which includes the NCD domain, has affinity for cell walls of *S*. *aureus* and *L*. *lactis*. In contrast, the C-terminal region does apparently not bind cell walls by itself. Yet, the highest levels of cell wall binding were observed for the intact IsaA protein, indicating that both the N- and C-regions are involved in cell wall binding with a potentially more prominent contribution from the N-region. It should be noted, however, that a substantial portion of IsaA is secreted into the growth medium, and this is also the case for SceD. Consistent with the observed cell wall binding of IsaA, we did observe localization of this protein to the *S*. *aureus* cell surface using an immunofluorescence microscopy approach. Of note, this involved an IRDye 800CW-labeled F(ab)_2_ fragment of the humAb 1D9, which binds to the N1 subdomain of IsaA. Together, these observations imply that the N-domain of IsaA is exposed on the *S*. *aureus* cell surface, where it is accessible to IgG.

Intriguingly, our present results show that sera of patients with the genetic blistering disease EB contain IgGs that predominantly target the N-domain of IsaA. Chronic wounds of these patients are heavily colonized with *S*. *aureus* ^43, 44^, which was previously shown to elicit IgG levels against *S*. *aureus* proteins that were much higher than the IgG levels against the respective proteins in healthy volunteers^23^. The highest IgG levels were detected against the IsaA protein, leading to the view that these IgGs could be protective against *S*. *aureus* infections^23^. This view is supported by the fact that EB patients rarely suffer from invasive *S*. *aureus* infections despite the high-level colonization of their wounds^45^, and by the observation that monoclonal antibodies against IsaA (i.e. 1D9 and UK-66) can be protective against *S*. *aureus* infection in mouse models^6–8^. Of note, our present study also shows that that the protective humAb 1D9 also recognizes the N-domain of IsaA. In contrast to our findings with the sera of EB patients, the immunization of mice with purified IsaA did not elicit the production of IgGs that protect against *S*. *aureus* infection. In this case, the IsaA-specific IgGs predominantly recognized the C-terminal region of IsaA as shown in our present study. Together, these observations imply that IgGs against the C-terminal region of IsaA do not effectively protect against *S*. *aureus* infection. On the other hand, it is presently not possible to conclude that the high titers of anti-IsaA antibodies in EB patients, which bind to the N-terminal domain of IsaA, are indeed protective. For example, antibodies against other *S*. *aureus* components that are also present at high levels could contribute to the protection of EB patients against *S*. *aureus* bacteremia. In addition, it is also conceivable that differences in cell-medicated immunity contribute to the apparent protection of EB patients against *S*. *aureus*.

A key question that remains to be answered is why does there appear to be a bias towards IgGs specific for the N-terminal region of IsaA in EB patient sera, and a bias towards IgGs specific for the C-terminal region in immunized mice? Our present studies show that the N-terminal region of IsaA is exposed on the cell surface of *S*. *aureus*, which might suggest that this domain is better exposed to the human immune system than the C-terminal region of IsaA. Yet, our previous proteomics study, where *S*. *aureus* cells were shaved with trypsin that was immobilized on agarose beads, clearly showed that also the C-terminus of IsaA is exposed to the *S*. *aureus* cell surface^12, 18^. This makes a differential exposure of the N- and C-terminal regions of IsaA on the *S*. *aureus* cell surface a less likely explanation for the observed differences in the IgG specificities from mice and men. An alternative explanation could be that the immunogenicity of the N- and C-terminal domains of IsaA is different, depending on whether the immune system is challenged with IsaA attached to the *S*. *aureus* cell surface or with soluble IsaA. Conceivably, such a difference could relate to conformational differences in the respective domains when they are associated with the cell or secreted into the growth medium.

## Conclusion

Altogether, our present study shows that the immune responses to IsaA are very different in the investigated mouse and human sera. In particular, the results seem to suggest that IgGs against the N-terminal domain of IsaA are potentially protective against *S*. *aureus* infection. This focuses attention on the N-terminal region of IsaA as a potential component in a future vaccine against the important human pathogen *S*. *aureus*. To validate this idea, protection studies need to be performed in an appropriate animal model, where the separated N- and C-terminal fragments of IsaA are used for active immunization.

## Electronic supplementary material

Supplementary Figures 1-3

Supplementary Table 1
