# Supplementary material for: Differential epitope recognition in the immunodominant staphylococcal antigen A of Staphylococcus aureus by mouse versus human IgG antibodies
Source: Sci Rep. 2017 Aug 15;7:8141. doi: 10.1038/s41598-017-08182-9 (PMC5557936; doi:10.1038/s41598-017-08182-9)
Supplement: Supplementary file 1 — Supplementary Figures 1-3 [file 41598_2017_8182_MOESM1_ESM.doc]

**Supplementary Material**

**Figures 1 - 3**

**Differential epitope recognition in the immunodominant staphylococcal antigen A of *Staphylococcus aureus* by mouse versus human IgG antibodies**

Danny G.A.M. Koedijk^1*^, Francisco Romero Pastrana^1*^, Hedzer Hoekstra^1^, Sanne van den Berg^2^, Jaap Willem Back^3^, Carolien Kerstholt^1^, Rianne C. Prins^1^, Irma A. J. M. Bakker-Woudenberg^2^, Jan Maarten van Dijl^1#^, and Girbe Buist^1^

^1^ Department of Medical Microbiology, University of Groningen, University Medical Center Groningen, Hanzeplein 1, P.O. Box 30001, 9700 RB Groningen, The Netherlands

^2^ Department of Medical Microbiology and Infectious Diseases, Erasmus University Medical Center, Rotterdam, The Netherlands

^3^ Pepscan Therapeutics BV, Lelystad, the Netherlands,

**^*^ These authors contributed equally.**

**Supplementary Figure 1**

**
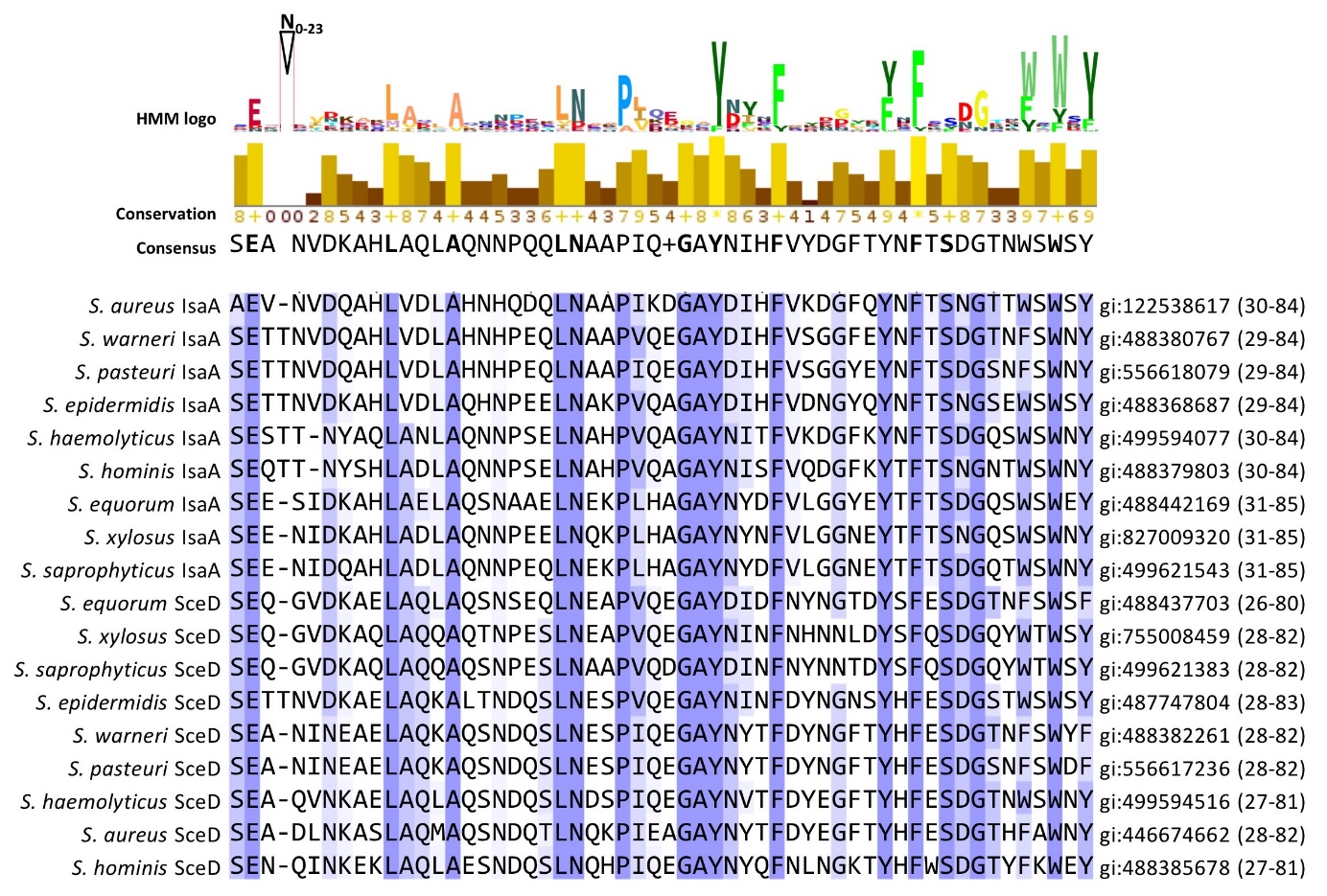
**

**Supplementary Figure 1. Conserved N-terminal domain of IsaA**

Conservation of amino acid residues in the ‘N-terminal conserved domain’ (NCD) of IsaA was assessed by multiple alignment of 391 protein reference sequences with residues 30-84 of IsaA. The alignment shows the similarity of the NCDs of the IsaA and SceD proteins from different staphylococcal species. Residues identical to the consensus sequence are marked in dark blue; residues with a positive Blosum62 score compared to the consensus are marked in light blue. The protein sequence gi numbers and the respective amino acid regions (in parentheses) are indicated.

**Supplementary Figure 2**

**Supplementary Figure 2. IgG titers in sera of mice immunized with IsaA**

Six mice were immunized subcutaneously with IsaA-His_6_ at days -28, -21 and -14 (25 μg), and 11 mice were placebo immunized following the same schedule. The IsaA-specific IgG titers on day -1 were assessed by ELISA. Each symbol represents a single mouse. The median values is indicated by a horizontal line.

**Supplementary Figure 3**


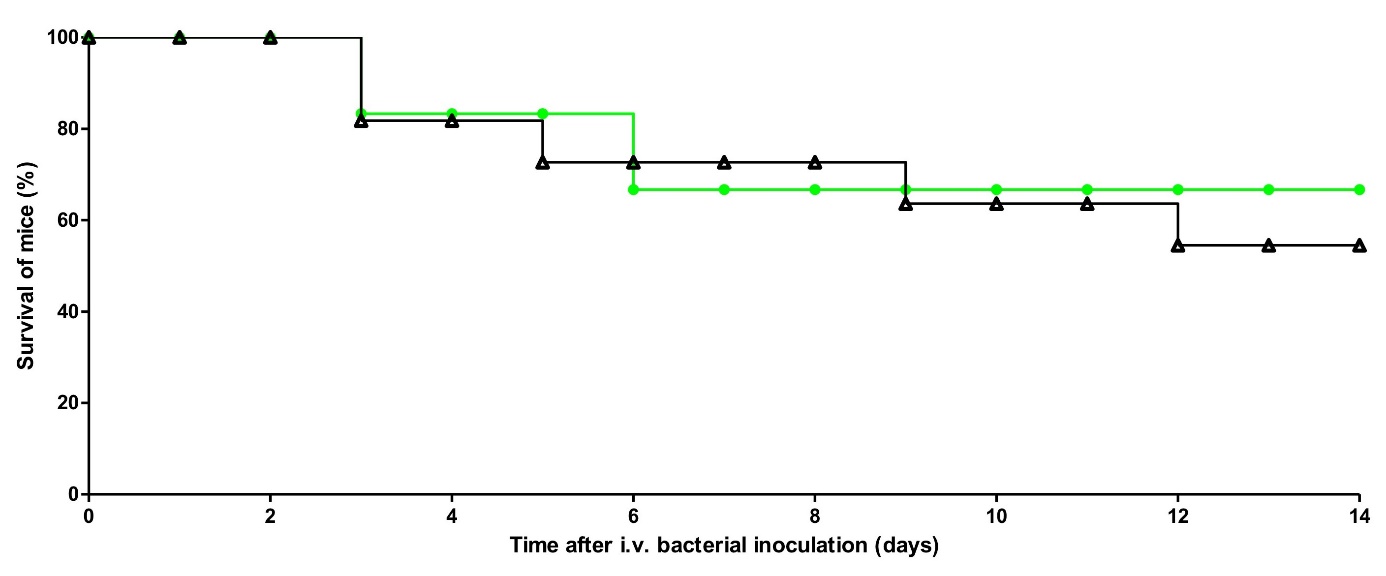


**Supplementary Figure 3. Survival of immunized mice with *S. aureus* isolate P bacteremia**

Mice immunized with IsaA (▲, n = 6) or placebo-immunized mice (●, n = 11) were infected with the clinical *S. aureus* isolate P by intravenous injection of 3 × 10^5^ colony-forming units (CFU). Subsequently, the infected mice were monitored for 14 days. No statistically significant difference in animal survival rates was observed (P > 0.05; log rank test).
