# Supplementary material for: Differential epitope recognition in the immunodominant staphylococcal antigen A of Staphylococcus aureus by mouse versus human IgG antibodies
Source: Sci Rep. 2017 Aug 15;7:8141. doi: 10.1038/s41598-017-08182-9 (PMC5557936; doi:10.1038/s41598-017-08182-9)
Supplement: Supplementary file 2 — Supplementary Table 1 [file 41598_2017_8182_MOESM2_ESM.doc]

**Supplementary Material**

**Table 1**

**Differential epitope recognition in the immunodominant staphylococcal antigen A of *Staphylococcus aureus* by mouse versus human IgG antibodies**

Danny G.A.M. Koedijk^1*^, Francisco Romero Pastrana^1*^, Hedzer Hoekstra^1^, Sanne van den Berg^2^, Jaap Willem Back^3^, Carolien Kerstholt^1^, Rianne C. Prins^1^, Irma A. J. M. Bakker-Woudenberg^2^, Jan Maarten van Dijl^1#^, and Girbe Buist^1^

^1^ Department of Medical Microbiology, University of Groningen, University Medical Center Groningen, Hanzeplein 1, P.O. Box 30001, 9700 RB Groningen, The Netherlands

^2^ Department of Medical Microbiology and Infectious Diseases, Erasmus University Medical Center, Rotterdam, The Netherlands

^3^ Pepscan Therapeutics BV, Lelystad, the Netherlands,

**^*^ These authors contributed equally.**

**Table 1. Primers used in PCR to express *isaA* gene fragments**

| Primer *^b^* | Sequence (5' > 3') *^a^* | Restriction site |
| --- | --- | --- |
| N1-F | ATATGGATCCGCTGAAGTAAACGTTGATCAAG | *Bam*HI |
| N2-R1 | ATATGCGGCCGCTGAACTTGAAGTAGTTGAAGTGCTGTAG | NotI |
| N2-F | ATATGGATCCGCTGGTTTCTCAAACGTTGC | *Bam*HI |
| N2-R2 | ATATGCGGCCGC**tta**TGAACTTGAAGTAGTTGAAGTGCTGTAG | *Not*I |
| C1-F | ATATGGATCCGTGAGATTAAGCAATGGTAATACTG | *Bam*HI |
| C1-R1 | ATATGCGGCCGCATTTACTTGACCATTTGATTCAC | *Not*I |
| C2-F | ATATGGATCCGCTTACAACCCATCAGGTGCTTCAG | *Bam*HI |
| C1-R2 | ATATGCGGCCGC**tta**ATTTACTTGACCATTTGATTCAC | *Not*I |
| N1-R1 | ATATGCGGCCGCAGTTTGACCATTAGCTGCTTCATAG | *Not*I |
| C2-R1 | ATATGCGGCCGCGAATCCCCAAGCACCTAAACCTTG | *Not*I |
| N1-R2 | ATATGCGGCCGC**tta**AGTTTGACCATTAGCTGCTTCATAG | *Not*I |
| C2-R2 | ATATGCGGCCGC**tta**GAATCCCCAAGCACCTAAACCTTG | *Not*I |

*^a^ Restriction site underlined, stop codon in lower case and bold; ^b^ Forward (F) and reverse (R1/R2) primer sequences are shown.*
